# Efficacy and Safety of Ultrasound-Guided Radiofrequency Treatment for Chronic Pain in Patients with Knee Osteoarthritis: A Systematic Review and Meta-Analysis

**DOI:** 10.1155/2020/2537075

**Published:** 2020-09-19

**Authors:** Yuzhao Huang, Qiufang Deng, Liuqing Yang, Jiahui Ma, Ziyang Wang, Dong Huang, Ling Luo, Haocheng Zhou

**Affiliations:** ^1^Department of Orthopaedics, The Third Xiangya Hospital, Central South University, Changsha, Hunan 410013, China; ^2^Department of Endocrinology, The Third Xiangya Hospital, Central South University, Changsha, Hunan 410013, China; ^3^Department of Pain, The Third Xiangya Hospital and Institute of Pain Medicine, Central South University, Changsha, Hunan 410013, China

## Abstract

**Background:**

Knee osteoarthritis (KOA) is a common degenerative disease associated with joint dysfunction and pain. Ultrasound-guided radiofrequency (RF) may be a promising therapy in the treatment of chronic pain for KOA patients.

**Objective:**

To evaluate the efficacy and safety of ultrasound-guided RF treatment for chronic pain in patients with KOA.

**Design:**

A systematic review was conducted, and a meta-analysis was carried out when possible. *Setting*. We examined the studies evaluating the clinical efficiency of ultrasound-guided RF on chronic pain in KOA population.

**Method:**

A systematic review for the efficacy and safety of ultrasound-guided RF treatment for pain management of KOA patients was carried out in PubMed, EMBASE, Cochrane Library, Web of Science, Wanfang Data, and China National Knowledge Infrastructure (CNKI) from the date of inception to February 2020, and a meta-analysis was conducted. The primary outcomes of pain intensity (visual analogue scale or numerical rating scale) and knee function [the Western Ontario and McMaster Universities Osteoarthritis Index (WOMAC)] were evaluated from baseline to various follow-up times by random-effects model. Heterogeneity was assessed by *I*^2^ statistic and the potential sources of heterogeneity by subgroup and metaregression analyses, respectively.

**Results:**

Eight publications with 256 patients were included in the meta-analysis. RF could relieve pain with −4.196 of pooled mean difference and improve knee function by decreasing 23.155 points in WOMAC. Three patients had ecchymosis, two with hypoesthesia and one with numbness after the procedure, and improved within 6 months. Furthermore, study design and treatment target were the sources of heterogeneity by subgroup and metaregression analyses, accounting for 37% and 74% of variances, respectively. Target of genicular nerve achieved better pain relief than intra-articular or sciatic nerve. Sensitivity analysis showed that removal of any single study was unlikely to overturn the findings. *Limitations*. There were some limitations in the study. Firstly, the small number of relevant studies limited the confidence level of the meta-analysis. Also, the significant heterogeneity may not be explained due to the limited data. Secondly, the direct comparison of two different guidance methods (ultrasound vs. fluoroscopy) for RF therapy is lacking. In addition, the outcomes were blindly assessed in the meta-analysis from all studies according to evaluation of bias, which could affect the reality of the data. Finally, most of the studies only provided short follow-up times, so we could not analyze the long-term effectiveness of ultrasound-guided RF in the treatment of patients with KOA.

**Conclusions:**

Ultrasonography is an effective, safe, nonradiative, and easily applicable guidance method for RF in pain relief and functional improvement in KOA patients.

## 1. Introduction

Knee osteoarthritis (KOA) is a very common joint disease and associated with diverse factors including age, obesity, metabolic bone diseases, acute or chronic joint injuries, etc. [1]. The prevalence of KOA ranges from 4.2% to 15.5% and gradually increases with age. Approximately 80% of KOA patients could be diagnosed by imaging at the age of 65 years or older, while only 60% of patients have shown clinical manifestations [2, 3]. Pain and disabilities are the major consequences of KOA, with 25% of patients suffering from severe arthralgia. Furthermore, KOA was ranked 11th among the 291 disabling illnesses worldwide [4]. It is currently believed that failure of chondrocytes to maintain homeostasis between synthesis and degradation of extracellular matrix and subchondral bone leads to osteoarthritis [5–8]. Treatments of KOA include noninvasive therapies such as medication, physical therapy, and rehabilitation as well as minimally invasive strategies from intra‐ or periarticular injections to radiofrequency (RF) [9]. Multiple studies have shown that postoperative RF therapy could accelerate the early rehabilitation of the joints in patients with late stage of KOA after joint replacement surgery [10, 11].

Recently, minimally invasive RF has been extensively used in the treatment of different stages of KOA and has achieved convincing therapeutic benefits. However, conventional RF is routinely guided by X-rays, so it may increase the risk of radiation exposure to the patients and health care providers [12]. Thus, musculoskeletal ultrasonography has become a potential guidance method for RF instead of fluoroscopy in chronic pain management due to its unique advantages [13, 14]. For example, ultrasound guidance is very accurate in peripheral or paraspinal nerve blocks to avoid injury of blood vessels and pleura [15, 16]. The efficacy of ultrasound-guided intervention is associated with many factors such as the settings of ultrasound device, preoperative administration of diagnostic nerve block (DNB), the location of targeted site, the skill of physician, etc. [17]. In recent years, more studies have demonstrated its therapeutic effects on the improvements of soreness, pain, and functional impairments induced by KOA, including case reports, retrospective and prospective uncontrolled studies, and randomized controlled trials (RCTs). However, confounding factors from these studies such as sample size, different methods or procedures may affect the outcomes. Meanwhile, there is no systematic analysis for evaluating the efficacy and safety of ultrasound-guided RF in the treatment of chronic pain in KOA patients. Therefore, we searched several databases from relevant literature to perform a systematic review and meta-analysis to evaluate the efficacy and safety of ultrasound-guided RF for providing preliminary scientific evidence for its clinical application in the treatment of patients with KOA.

## 2. Methods

### 2.1. Design

A systematic review was conducted, and a meta-analysis was carried out when possible.

### 2.2. Search Strategy

We systematically searched several electronic databases including PubMed, Excerpta Medica Database (EMBASE), Cochrane Library, Web of Science, Wanfang Data, and China National Knowledge Infrastructure (CNKI) via strategies developed using the appropriate Medical Subject Headings (MeSH) terms from the date of inception to February 2020. Keywords such as “knee osteoarthritis,” “ultrasound guided,” “radiofrequency therapy,” “genicular nerve,” “intra-articular,” and “chronic knee pain” were used. No date, language, or country limitations were applied to the searching.

### 2.3. Inclusion Criteria

The inclusion criteria for the meta-analysis were as follows: (1) human clinical trials with or without control groups and cointervention were allowed if the trial was performed equally to both arms; (2) patients were diagnosed with KOA and suffered from chronic pain without satisfying pain relief by conservative therapies; (3) patients received RF therapy such as pulsed radiofrequency (PRF) or radiofrequency ablation (RFA); (4) minimally invasive procedure was completed under the guidance of ultrasound; and (5) necessary evaluation index was provided before and after RF therapy, for pain intensity and knee function including visual analogue scale (VAS), numerical rating scale (NRS), Oxford Knee Score (OKS), or Western Ontario and McMaster Universities Osteoarthritis Index (WOMAC) and for quality of life including 36-Item Short-Form Health Survey (SF-36).

### 2.4. Exclusion Criteria

The exclusion criteria for the meta-analysis were as follows: (1) full text is not available; (2) patients received total knee arthroplasty (TKA) or other knee surgery; (3) case report; (4) studies with insufficient data or uncompleted RCT; and (5) studies with doubtful data such as illogical outcomes without reasonable explanation.

### 2.5. Study Selection

After targeted publications were found from different databases, the duplicates were removed first by two experienced investigators independently. Next, irrelevant studies were excluded by further scanning the title and abstract of publication by the inclusion criteria, and then the full text of remaining study was carefully screened to identify eligibility according to the exclusion criteria. Any uncertainty or disagreements were finally resolved via discussion between the two investigators and consulted with the third investigator to reach consensus.

### 2.6. Data Extraction

Two investigators independently extracted relevant data from each study including the first and corresponding authors, year of publication, country, study design, sample size, demographic characteristics (age and gender), grade of radiologic KOA (Kellgren–Lawrence grading system), follow-up time, type of RF, ultrasound transducer parameter, treatment targets and controls, primary outcomes such as the scores of pain intensity (VAS or NRS) and knee function (WOMAC or Lysholm knee scoring scale) at baseline and available follow-up times, complications or adverse effects, conclusion, and limitations. We contacted the first and/or corresponding authors of study to verify any unclear information and data by e-mails, and the data were considered to be irretrievable without a reply from the authors. All the information was recorded in a prepared spreadsheet, and data were fully analyzed after collection.

### 2.7. Quality Assessment

The quality and risk of bias for each study were independently assessed by at least two examiners. Additional investigators were consulted when discrepancies were present. RCTs were assessed by the criteria from the Cochrane Handbook for Systematic Reviews of Interventions [18]. The potential sources of bias include random sequence generation (selection bias), allocation concealment (selection bias), blinding of participants and personnel (performance bias), blinding of outcome assessment (detection bias), incomplete outcome data (attrition bias), selective reporting (reporting bias), and other bias were judged as “low risk,” “high risk,” or “unclear risk,” respectively. For nonrandomized studies, different biases were determined by the criteria according to “Assessing the Risk of Bias of Individual Studies in Systematic Reviews of Health Care Interventions” [19]. This specific form contains 9 questions, and each question represents a potential source of bias. Positive answer indicates low risk of bias, while negative answer means high risk of bias. Newcastle-Ottawa Scale (NOS) criteria were also used for reference [20].

### 2.8. Statistical Analysis

One of the primary outcomes from the studies was the pain intensity of patients as reported as the VAS (0–10 or 0–100 mm) or NRS (0–10) in different studies. To standardize the pain scale, the VAS (0–10 cm) was equivalent to the NRS (0–10) and transformed the scale from 0–10 cm to 0–100 mm. The 95% confidence interval (CI) for the difference in means was used to measure the scores of pain and knee function (WOMAC). For each analysis, the heterogeneity test was performed with *I*^2^ statistics to measure the degree of data inconsistency as *I*^2^ > 50% being statistically significant between studies. Data were also analyzed with the random-effects model for high heterogeneity. Subgroup analysis was conducted for study design (RCT vs. prospective vs. retrospective study), treatment target (intra-articular vs. genicular vs. sciatic nerve), the performance of DNB, and follow-up period (0 vs. 4, 12, or 24 weeks). Metaregression analyses were performed to evaluate the sources of heterogeneity based on all the covariates including age and gender in subgroup analysis. Sensitivity analysis was conducted to evaluate the impact of every single study on the pooled mean difference (MD). In addition, the publication bias was evaluated by Begg and Mazumdar rank correlation test and Egger's regression test [21, 22]. Comprehensive meta-analysis (CMA version 3.0, Biostat, Englewood, NJ, USA) was used to analyze the pooled data.

## 3. Results

### 3.1. Study Selection

**Figure 1:**
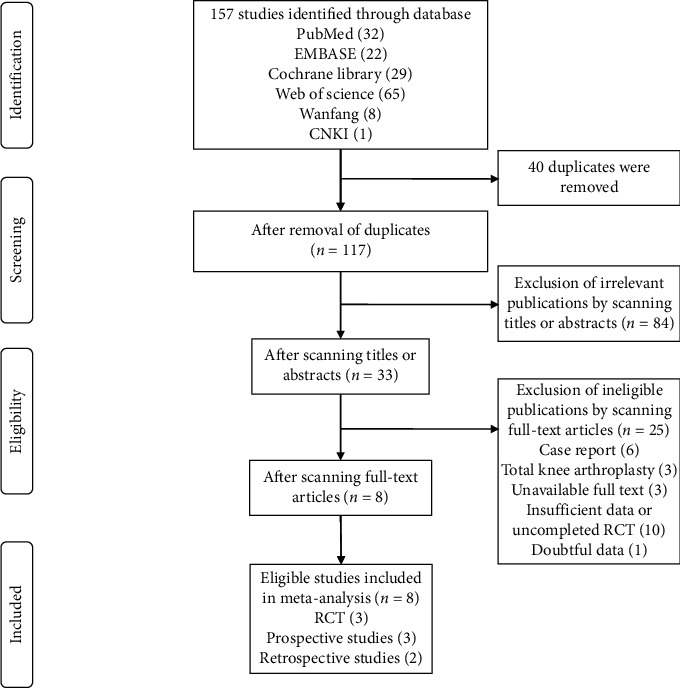
Flowchart of study selection.

A total of 157 publications were identified from six electronic databases and 117 studies for further screening after removing 40 duplicates. Eighty-four irrelevant studies were removed through screening the titles and abstracts of publications, and 25 additional studies were excluded by exclusion criteria via full-text screening. Finally, eight eligible publications were included in the study of meta-analysis including 3 RCTs, 3 prospective trials, and 2 retrospective studies [23–30]. The screening method and results of the relevant studies are illustrated in Figure 1.

### 3.2. Study Characteristics

The included studies were conducted in five countries including Spain 3, Turkey 2, Egypt 1, India 1, and China 1, and the published date was from 2013 to 2019. The studies had 256 patients in total, with 61 males and 195 females, and the mean ages ranged from 60.0 to 72.5 years. The characteristics of studies were presented in Table 1. For RF therapy, PRF was used in 4 and RFA in 3 studies, while the combination of PRF and RFA was used in one study [25]. Most studies of RF therapy were focused on sciatic nerve or genicular nerve, but two studies applied intra-articular procedure [24, 27]. Furthermore, DNB was used to confirm the source of pain and positioning targets of RF therapy in 2 studies [27, 30]. VAS/NRS scores were available to compare the changes of pain intensity before and after RF therapy in 7 studies. In addition, WOMAC and Lysholm scores were available to evaluate the functional improvement from baseline to various follow-up times in 7 studies. The most of follow-up times were up to half year (0, 4, 12, and 24 weeks), and only one study was followed up to one year (0, 4, 12, 24, and 48 weeks). The ultrasound transducer parameter, complication or adverse effect, conclusion and limitation of studies are presented in Tables 2 and 3.

### 3.3. Clinical Outcomes

**Figure 2:**
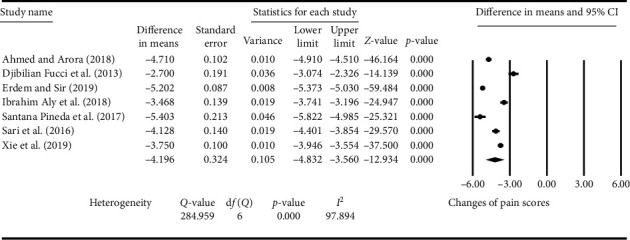
Summary of different biases in the randomized controlled trials (RCTs) of ultrasound-guided radiofrequency (RF) in the treatment of patients with knee osteoarthritis (KOA).

**Figure 3:**
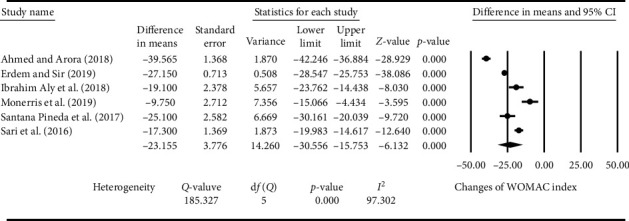
Percentage (%) of the risk of bias in the randomized controlled trials (RCTs) of ultrasound-guided radiofrequency (RF) in the treatment of patients with knee osteoarthritis (KOA).

The primary clinical outcomes for ultrasound-guided RF therapy were pain relief and functional improvement in patients with OA, and the results are shown in Figures 2 and 3. Significant pain relief was achieved by the treatment of ultrasound-guided RF in 7 studies [23, 24, 26–30], and the pooled mean difference of pain score was −4.196 (SE: 0.324; 95% CI: −4.832 to −3.560; *P* < 0.001; *I*^2^: 97.894%) compared to that of pretreatment (baseline) in patients with OA (Figure 2).

As shown in in Figure 3, knee function was also significantly improved after the treatment of ultrasound-guided RF in patients with OA in six studies [23, 25, 27–30]. WOMAC was decreased by 23.155 points (SE: 3.776; 95% CI: −30.556 to −15.753; *P* < 0.001; *I*^2^: 97.302%) after the treatment of ultrasound-guided RF compared to that of baseline in patients with OA.

### 3.4. Adverse Effect

Ultrasound-guided RF induced adverse events were uncommon and not serious; 3 patients were reported with ecchymosis at the site of procedure in the study by Santana Pineda et al. [28] and two patients with hypoesthesia and one patient with numbness in the study by Ahmed and Arora [30] after the therapy, and all the symptoms were improved by more than 50% within 6 months of treatment. No other complications have been reported in the patients with ultrasound-guided RF therapy. No adverse event was even reported in other 6 studies.

### 3.5. Risk of Bias

**Figure 4:**
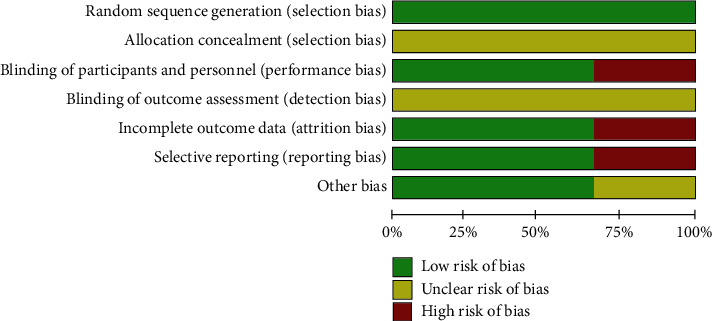
Effect of ultrasound-guided radiofrequency (RF) on the pain scores in patients with knee osteoarthritis (KOA). VAS: visual analogue scale; NRS: numerical rating scale.

**Figure 5:**
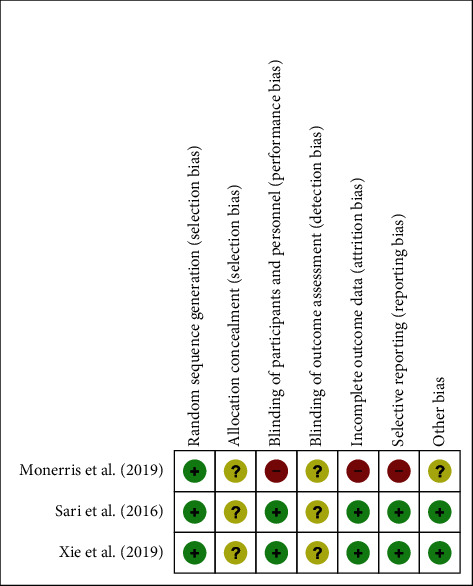
Effect of ultrasound-guided radiofrequency (RF) on the Western Ontario and McMaster Universities Osteoarthritis Index (WOMAC).

As mentioned previously, two different methods were used to evaluate the risk of bias. For RCTs, the risks of selection, performance, attrition, report, detection, and other bias were determined by the criteria from the Cochrane Handbook for Systematic Reviews of Interventions [19]. The risks of allocation concealment (selection bias) and blinding of outcome assessment (detection bias) were unclear in all three RCTs. However, the study by Monerris and colleagues [25] had 3 potential sources of bias, indicating at high risk. The risks of biases are summarized in Figures 4 and 5 for RCTs. For nonrandomized clinical trials, a design-specific form containing nine questions was used to determine bias according to “Assessing the Risk of Bias of Individual Studies in Systematic Reviews of Health Care Interventions” [20]. Particularly, there was not enough information for evaluating blinding of outcome assessment in most of nonrandomized trials. The detailed risks of bias in each study are presented in Table 4.

### 3.6. Publication Bias

No publication bias was found for the primary outcomes (pain relief and functional improvement) of RF therapy in patients with OA. The results showed *P*=0.332 and *P*=0.274 for pain relief and *P*=0.245 and *P*=0.226 for functional improvement (by Egger's regression test and Begg and Mazumdar rank correlation test, resp.).

### 3.7. Subgroup Analysis and Metaregression Analysis

Subgroup analysis was performed to confirm the sources of heterogeneity for pain intensity associated with study design (RCTs vs. prospective vs. retrospective studies), treatment targets (intra-articular vs. genicular vs. sciatic nerves), administration of DNB before treatment (applied vs. unapplied DNB), and time of follow-up (0 vs. 4, 12, or 24 weeks), and the results are presented in Table 5. The data from subgroup analysis showed that study design (RCTs MD: −3.926, 95% CI: −4.296 to −3.557; prospective MD: −3.853; 95% CI −5.241 to −2.464; and retrospective MD: −4.959; 95% CI: −5.440 to −4.447) and treatment targets (intra-articular MD: −3.626; 95% CI: −3.900 to −3.352; genicular nerve MD: −4.851; 95% CI: −5.350 to −4.452; and sciatic nerve MD: −2.700; 95% CI −3.074 to −2.326) were the potential sources of heterogeneity (*P* < 0.01 and *P* < 0.001, resp.). However, there was no significant difference in pain relief whether DNB was administrated or not before RF therapy and among different follow-up periods (*P* > 0.05).

**Figure 6:**
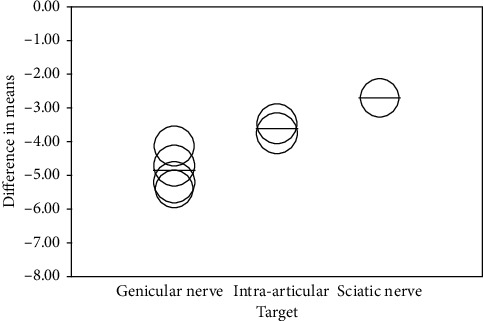
The correlation between treatment target and the pain scores in patients with knee osteoarthritis (KOA) by metaregression.

Furthermore, we also performed a metaregression analysis based on all the covariates in subgroup analysis including age and gender to verify the sources of heterogeneity, and the results are shown in Table 6 and Figure 6. The data revealed that different study designs accounted for 37% and different treatment targets for 74% in pain relief between-study variance. Target of genicular nerve (GN) achieved best pain relief while sciatic nerve was the least effective target among the 3 nerves. However, other covariates may not account for any heterogeneity according to the analysis.

### 3.8. Sensitivity Analysis and Other Evaluation Indices

**Figure 7:**
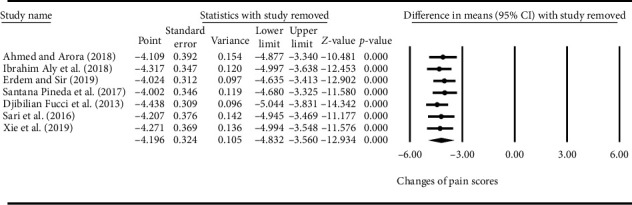
Effect of ultrasound-guided radiofrequency (RF) on pain scores by sensitivity analysis.

The results in Figure 7 exhibited the stability of pooled effect size via sensitivity analysis of pain scores. The data showed that the conclusion of meta-analysis could not be overturned by removing any single study; besides, the names of studies were recalculated with pooled MD after removal of each study.

Moreover, the total time spent on the procedure for ultrasound-guided RF was recorded and compared to that of fluoroscopy-guided RF in the study by Sarı and colleagues [23]. The duration was 20.2 ± 6.4 min for ultrasonography and 25.0 ± 4.8 min for fluoroscopy, respectively. The data indicate that performance of ultrasound-guided RF requires much less time than that of fluoroscopy-guided RF. For knee functional improvement, Xie et al. reported that the Lysholm scores were increased from pretreatment of 53 ± 9 (baseline) to 79 ± 7 (4 weeks) and 70 ± 8 (24 weeks) after RF therapy, and SF-36 scores were also improved from pretreatment of 407 ± 91 (baseline) to 597 ± 102 (4 weeks) and 541 ± 95 (24 weeks), respectively; there were statistically significant differences (*P* < 0.01) before and after RF therapy for both Lysholm and SF-36 scores [22]. In addition, Likert scale was used to assess patient's satisfaction in the studies by Santana Pineda et al. and Erdem and Sir [29, 30]. Santana Pineda et al. reported that the scores were poor in 2 (2/25), average in 1 (1/25), good in 5 (5/25), and very good in 16 (16/25) in total of 25 patients after 24 weeks of RF therapy. In the study by Erdem and Sir, the outcomes of ultrasound-guided RF therapy were uncertain in 3 (3/17), good in 3 (3/17), and very good in 11 (11/17) in total of 17 treated patients. Both studies indicate that most of the patients were significantly improved by ultrasound-guided RF therapy. Furthermore, the study by Ahmed and Arora also reported significant improvement in pain intensity and the quality of life after RF therapy (*P* < 0.05) [4]. The OKS and WOMAC were improved from 7.75 ± 1.25 and 77.75 ± 4.34 at baseline to 28.88 ± 2.53 and 38.38 ± 5.82 at 4 weeks, 28.13 ± 1.80 and 39.25 ± 5.12 at 24 weeks of therapy, respectively.

## 4. Discussion

KOA is a very common disease and has become a huge economic burden on our society [1, 4]. Patients with KOA suffer intractable pain with high risk of disability. Pain management plays a major role in the treatment of KOA for pain relief and knee function improvement [31]. Different treatments are applied to different patients based on the severity of KOA. Generally, conservative therapies and TKA are commonly used treatments for KOA. However, some patients are unwilling to or could not tolerate TKA while conservative therapies could not achieve satisfying pain relief. Therefore, more effective and safe therapeutic strategy is urgently needed for pain relief in the patients with KOA.

Recently, RF has been widely used to relieve intractable pain in KOA patients as a novel minimal invasion technique [32, 33]. Choi et al. first reported the efficacy of RF to relieve pain by targeting genicular nerve in patients with chronic KOA from a double-blind, randomized controlled trial [12]. Fluoroscopy is the most used method to guide RF currently. However, more and more cases were registered for clinical trials with ultrasound-guided RF therapy in patients with OA recently [34], indicating that ultrasound may have some unique advantages and could be a potential guidance method in place of fluoroscopy.

### 4.1. Summary of the Main Results

In the study of meta-analysis, 8 articles with a total of 256 patients were analyzed to evaluate the effect of ultrasound-guided RF on pain relief and knee functional recovery in patients with KOA. The main results revealed that all the patients suffered from intractable knee pain before treatment, and pain intensity and knee function were significantly improved from baseline (pretreatment) to different follow-up times after RF therapy. In an RCT for comparison of the efficacy of ultrasound- and fluoroscopy-guided RF in KOA patients by Sari et al. [23], ultrasound-guided RF achieved the same therapeutic effects as those of fluoroscopy-guided RF for pain relief and functional improvement, but the procedure time was significantly less than that of fluoroscopy. Furthermore, the incidence of adverse events is very low (2.33%) after ultrasound-guided RF therapy, and only 6 patients experienced adverse events in 3 patients with ecchymosis at the site of procedure [28], two patients with hypoesthesia [30], and one patient with numbness [30] from 256 treated patients, and these symptoms were significantly improved or disappeared in the next 6 months. No adverse event was even reported in other 6 studies.

For the meta-analysis, the patients with previous TKA used only as control were excluded from the calculation according to our exclusion criteria in the study by Erdem and Sir [29]. In the subgroup analysis, we studied the changes of pain intensity with different study design (RCT vs. prospective vs. retrospective), treatment targets (intra-articular vs. genicular vs. sciatic nerves), with or without DNB, and duration of follow-up (0 vs. 4, 12, or 24 weeks). The results from metaregression analysis revealed that study design and treatment target would account for the major sources of heterogeneity as 37% and 74%, respectively. Furthermore, significant difference of pain relief was observed with different treatment targets by subgroup analysis, which showed that target of genicular nerves achieved better effect on pain relief than targeting intra-articular and sciatic nerves. The result suggests that genicular nerve is a preferable target in ultrasound-guided RF therapy in KOA patients. No significant difference of pain relief was observed with DNB and duration of follow-up by subgroup analysis (Table 6). However, pain scores were decreased in all of follow-up ties, indicating that the efficacy of ultrasound-guided RF therapy on KOA patients would maintain for at least 24 weeks. The data suggest that ultrasound-guided RF as a minimally invasive procedure could significantly relieve pain and improve knee function and it is effective, safe, and time-saving in the treatment of patients with KOA.

### 4.2. Ultrasound-Guided Therapy

The application of ultrasonography is rapidly increased for guidance of RF in pain management and knee function improvement in KOA patients. Over the last few years, more and more patients with KOA have used ultrasonography instead of fluoroscopy in RF therapy. Perrine et al. found that ultrasound- and fluoroscopy-guided RF therapy achieved the same effects on pain relief and functional improvement in KOA patients [32]. Kim et al. reported that ultrasound- and fluoroscopy-guided genicular nerve block also had similar effects on pain relief, functional improvement, and safety in patients with chronic KOA [35]. These studies suggested that RF could reach accurate localization of genicular nerve by ultrasonic guidance as by fluoroscopy. The sources of sensory nerve played the key role in knee pain and could help the operator to identify the appropriate branches of nerve for RF therapy with anatomic landmarks [36–38]. The studies by Ergonenc and Beyaz [39] and Wu et al. [40]. have reported the effect of ultrasound-guided RF therapy on targeting suprascapular nerve for the treatment of chronic shoulder pain, indicating that ultrasound-guided RF therapy could also be applied to treat other diseases such as musculoskeletal pain. Narouze reviewed the role of ultrasonography in spine interventional procedures in pain management from evidence-based studies [41] and demonstrated the effectiveness and safety of ultrasonography in RF therapy. Ultrasonography has several advantages over fluoroscopy. Firstly, ultrasonic guidance can be performed as a dynamic examination, and various tissues and arteries can be directly visualized under ultrasonography to help identifying the nerves and real-time needle advancement. In addition, the machine of ultrasound is more affordable and moveable than a fluoroscopy. Furthermore, the most important advantage of ultrasonography is radiation free, and prolonged or repeated exposure to radiation is harmful to the health care providers and patients [42]. Therefore, ultrasonography is apparently a better choice than fluoroscopy in RF therapy.

### 4.3. Limitations

There were some limitations in the study. First of all, we have only 8 enrolled studies with 256 patients for the meta-analysis. The small number of relevant studies and enrolled patients limited the confidence level of the meta-analysis. Secondly, another major problem is lacking high-quality RCTs to directly compare the two different guidance methods (ultrasound vs. fluoroscopy) for RF therapy in patients with KOA. Thirdly, high heterogeneity was observed in the studies. For example, study design and treatment targets were of the major between-study variances as accounted for 37% and 74% of variances, respectively; and *I*^2^ was more than 50 by subgroup analysis and metaregression analysis for potential sources of heterogeneity. In addition, the outcomes were blindly assessed in the meta-analysis from all studies according to evaluation of bias, which could affect the reality of the data. Finally, most of the studies only provided short follow-up times, so we could not analyze the long-term effectiveness of ultrasound-guided RF in the treatment of patients with KOA.

## 5. Conclusion

Although there were some limitations in the studies, the results still provided clear evidence that ultrasound-guided RF therapy was effective and safe in the treatment of KOA patients for pain relief and knee function improvement. Numerous uncompleted RCTs related to the meta-analysis were found from the Cochrane Library, indicating that researchers and physicians have paid more attention to the application of this novel technique. However, there are still some questions needed to be answered. Ultrasound-guided RF therapy to target genicular nerve has only been widely used in recent few years. There are no detailed criteria or recommendations for this procedure such as RF type and treatment targets currently. The long-term effectiveness of ultrasound-guided RF in the treatment of KOA is still needed to be defined due to limited data. Nevertheless, ultrasonography is an effective, safe, dynamic, easily applicable, and nonradiative guidance method for RF therapy to achieve satisfying pain relief and functional improvement in KOA patients who failed conservative treatment. However, the efficacy and safety of ultrasound-guided RF in the treatment of KOA requires further investigation for clinical validation by high-quality multicentric, randomized controlled trials with large sample size.

## Figures and Tables

**Table 1:** Included studies.

Study	Country	Type of study	Sample size	Gender (M/F)	Age mean (SD) (intervention/control)	K-L grade	Follow-up time (week)
Sari et al. [23]	Turkey	RCT	50	6/44	66.08 (10.52)/65.92 (8.71)	2-4	4, 12
Xie et al. [24]	China	RCT	54	23/31	60 (6)/59 (6)	2-3	4, 24
Monerris et al. [25]	Spain	RCT	25	7/18	75.2 (9.1)	3-4	4, 12, 24
Djibilian Fucci et al. [26]	Spain	Prospective study	47	6/41	70.6 (9.7)	—	4
Ibrahim Aly et al. [27]	Egypt	Prospective study	30	6/24	60.8 (7.1)	2-3	1, 4, 12
Santana Pineda et al. [28]	Spain	Prospective study	25	3/22	72.5 (9)	3-4	4, 24, 48
Erdem and Sir [29]	Turkey	Retrospective study	17	5/12	69.75 (11.82)	3-4	3, 12
Ahmed and Arora [30]	India	Retrospective study	8	5/3	65.75 (6.96)	3-4	4, 24

K-L grade: Kellgren–Lawrence grading system; RCT: randomized controlled trial.

**Table 2:** Details of intervention, evaluation criterion, adverse effect, conclusion, and limitation of the 3 RCTs.

Study	Intervention	Control	RF mode	Target location	Ultrasound transducer parameter	Diagnostic nerve block	Evaluation criterion	Reported adverse effect	Conclusion	Limitation
Sari et al. [23]	Ultrasound-guided RF	Fluoroscopic-guided RF	RFA	SM, SL, IM genicular nerves	8–14 MHz	No	VAS; WOMAC	None	Ultrasound-guided RF achieved same clinical efficacy but easily applicable, safe, dynamic, and no radiation compared to fluoroscopic-guided RF	Small sample size; short follow-up time

Xie et al. [24]	Acupotomy combined with RF	Acupotomy	PRF	Intra-articular	7–12 MHz	No	VAS; Lysholm; SF-36	None	Ultrasound-guided PRF combined with acupotomy has better clinical efficacy than acupotomy alone	Small sample size

Monerris et al. [25]	Ultrasound-guided RF	Sham RF treatment	PRF + RFA	PFA: saphenous nerve; RFA: SL, IL, IM genicular nerves	6–13 MHz	No	VAS; WOMAC; PGIC; SF-12	None	The combination of PRF and RFA did not achieve better therapeutic efficacy on knee pain and function compared to control	Small sample size; imperfect study design and data presentation

RF: radiofrequency; RFA: radiofrequency ablation; PRF: pulsed radiofrequency; SM: superior medial; SL: superior lateral; IM: inferior medial; IL: inferior lateral; VAS: visual analogue scale; WOMAC: Western Ontario and McMaster Universities Osteoarthritis Index; SF-36: 36-Item Short-Form Health Survey; PGIC: Patients' Global Impression of Change questionnaire; and SF-12: 12-Item Short-Form Health Survey.

**Table 3:** Details of intervention, evaluation criterion, adverse effects, conclusion, and limitation of the nonrandomized studies.

First author (year)	RF mode	Target location	Ultrasound transducer parameter	Diagnostic nerve block	Evaluation criterion	Reported adverse effects	Conclusion	Limitation
Djibilian Fucci et al. [26]	PRF	Sciatic nerve	3–6 MHz	No	VAS	None	Ultrasound-guided PRF on sciatic nerve significantly relieved pain and may become a novel therapeutic approach for chronic knee pain	Lack of control group; small sample size; short follow-up time; lack of evaluation for knee function

Ibrahim Aly et al. [27]	PRF	Intra-articular	6–13 MHz	Yes	NRS; WOMAC	Ecchymosis at the site of injection (3/30)	Intra-articular PRF was safe and beneficial for pain relief in patients with KOA	Lack of control; small sample size

Santana Pineda et al. [28]	RFA	SL, SM, IM genicular nerve	5–10 MHz	No	VAS; WOMAC	None	Ultrasound-guided RFA of genicular nerve was a safe, effective, minimally invasive treatment for chronic pain and disability induced by KOA	Lack of control group; small sample size

Erdem and Sir [29]	PRF	SL, SM, IM genicular nerve	6–15 MHz	No	VAS; WOMAC	None	Ultrasound-guided PRF targeting genicular nerves was a safe and minimally invasive procedure that significantly alleviated pain and disability in patients with severe KOA	Lack of control; small sample size; short follow-up time

Ahmed and Arora [30]	RFA	SM, SL, M, IM, IL, P genicular nerve; LRN	6–13 MHz	YES	NRS; OKS; WOMAC; SF-36	Hypoesthesia (2/8); numbness (1/8)	Ultrasound-guided RFA targeting genicular nerves was safe and effective for significantly improving pain, disability and quality of life in patients with severe KOA	Lack of control; small sample size

RF: radiofrequency; RFA: radiofrequency ablation; PRF: pulsed radiofrequency; SM: superior medial; SL: superior lateral; IM: inferior medial; IL: inferior lateral; M: middle; P: posterior; LRN: lateral retinacular nerve; VAS: visual analogue scale; NRS: numerical rating scale; WOMAC: Western Ontario and McMaster Universities Osteoarthritis Index; OKS: Oxford Knee Score; and SF-36: 36-Item Short-Form Health Survey.

**Table 4:** Evaluation of bias for nonrandomized studies.

Risk of bias criterion	Criterion	Djibilian Fucci et al. [26]	Ibrahim Aly et al. [27]	Santana Pineda et al. [28]	Erdem et al. [29]	Ahmed and Arora [30]
Selection bias	Does the design or analysis control account for important confounding and modifying variables through matching, stratification, multivariable analysis, or other approaches?	✗	✓	✓	✓	✓

Performance bias	Did researchers rule out any impact from a concurrent intervention or an unintended exposure that might bias results?	✓	✓	✓	✗	✗
	Did the study maintain fidelity to the intervention protocol?	✓	✓	✓	✓	✓

Attrition bias	If attrition (overall or differential nonresponse, dropout, loss to follow-up, or exclusion of participants) was a concern, were missing data handled appropriately (e.g., intention-to-treat analysis and imputation)?	✓	✓	✓	✓	✓

Detection bias	Were the outcome assessors blinded to the intervention or exposure status of participants?	—	—	—	✗	✗
	Were interventions/exposures assessed/defined using valid and reliable measures implemented consistently across all study participants?	✓	✓	✓	✓	✓
	Were outcomes assessed/defined using valid and reliable measures implemented consistently across all study participants?	✓	✓	✓	✓	✓
	Were confounding variables assessed using valid and reliable measures implemented consistently across all study participants?	✗	✓	✓	✓	✓

Reporting bias	Were the potential outcomes prespecified by the researchers? Were all prespecified outcomes reported?	✓	✓	✓	✓	✓

**Table 5:** The potential sources of heterogeneity on pain intensity by subgroup analysis.

Subgroup	Study number	Mean difference (95% CI)	*I* ^2^	*P* value
*Study design*				
RCT	2	−3.926 (−4.296 to −3.557)	79.334	0.003
Retrospective	2	−4.959 (−5.440 to −4.447)	92.529	
Prospective	3	−3.853 (−5.241 to −2.464)	97.870	

*Treatment target*				
IA	2	−3.626 (−3.900 to −3.352)	63.051	<0.001
GN	4	−4.851 (−5.350 to −4.352)	94.158	
SN	1	−2.700 (−3.074 to −2.326)	0	

*Diagnosis nerve block (DNB)*				
DNB	2	−4.093 (−5.309 to −2.876)	98.071	0.850
No DNB	5	−4.237 (−5.104 to −3.370)	98.272	

*Follow-up time (week)*				
4	7	−4.378 (−5.149 to −3.607)	97.484	0.820
12	3	−4.115 (−5.093 to −3.138)	96.229	
24	3	−4.172 (−4.728 to −3.617)	82.941	

IA: intra-articular; GN: genicular nerve; and SN: sciatic nerve.

**Table 6:** The sources of between-study heterogeneity on pain intensity by metaregression analysis.

Subgroup	*Q*-value	d*f*	*P* value	Proportion of variance by covariate
Age	0.68	1	0.411	0.02
Gender (ratio)	0.30	1	0.586	0
Study design	3.59	2	0.166	0.37
Treatment target	21.82	2	<0.001	0.74
Diagnosis nerve block (DNB)	0.03	1	0.857	0
Follow-up time	0.19	2	0.911	0

## Data Availability

There were no data used other than the original one collected for the objective of this study.
